# “Paradoxical” prognostic role of the TyG index and a novel machine learning-derived nomogram for colorectal cancer liver metastases

**DOI:** 10.3389/fnut.2026.1842975

**Published:** 2026-07-13

**Authors:** Taijun Yi, Xingyu Li, Zhu Lin, Zejin Lin, Yongling Liang, Huilin Jin, Yunle Wan, Guolin Li

**Affiliations:** 1 Department of General Surgery (Hepatobiliary, Pancreatic and Splenic Surgery), The Sixth Affiliated Hospital, Sun Yat-sen University, Guangzhou, Guangdong, China; 2 The Sixth Affiliated Hospital, Biomedical Innovation Center, Sun Yat-sen University, Guangzhou, Guangdong, China; 3 Department of General Surgery, Tongren Hospital, Shanghai Jiao Tong University School of Medicine, Shanghai, China

**Keywords:** colorectal cancer liver metastases, machine learning, predictive model, prognosis, TyG index

## Abstract

**Background:**

The triglyceride-glucose (TyG) index is a reliable surrogate marker of insulin resistance. However, its prognostic significance in patients with colorectal cancer liver metastases (CRLM) remains unclear. This study aimed to investigate its prognostic value and develop a machine learning (ML) - based predictive model in CRLM.

**Methods:**

A total of 644 patients with synchronous CRLM who underwent curative-intent treatment were enrolled in this single-center retrospective study. The association between the TyG index and overall survival (OS) was assessed using Kaplan-Meier analysis and Cox regression models. Key predictive variables were selected via LASSO regression and six ML algorithms, which were then utilized to construct a nomogram. The model’s performance was evaluated by the C-index and the area under the receiver operating characteristic curve (AUC), and compared against classical clinical risk scores (CRS).

**Results:**

The research revealed an inverse association between TyG index and CRLM. Multiple analyses confirmed that a low TyG index was an independent risk factor for shortened OS. ML methods identified nine key variables, including the TyG index, for nomogram construction. The nomogram demonstrated improved predictive performance in individualized prognosis prediction compared to the CRS: C-index 0.697 (95% CI: 0.627–0.767) vs. 0.631 (95% CI: 0.559–0.703); 5-year AUC 0.829 (95% CI: 0.755–0.904) vs. 0.741 (95% CI: 0.646–0.836).

**Conclusion:**

Unlike what was observed in previous studies, a low TyG index was associated with poor prognosis in CRLM. The ML-derived predictive model showed potential for OS prediction and clinical application, but further external validation is required.

## 1 Introduction

Colorectal cancer (CRC) remains a leading cause of cancer-related morbidity and mortality worldwide, with the liver representing the most frequent site of distant metastasis (1, 2). Although comprehensive treatment strategies centered on curative-intent surgery have markedly improved outcomes for patients with colorectal cancer liver metastases (CRLM), substantial heterogeneity in postoperative survival persists. Therefore, identifying reliable biomarkers for precise prognostic assessment is essential to guide individualized treatment and improve clinical outcomes.

Insulin resistance (IR), a hallmark of metabolic syndrome (3), is implicated in a spectrum of diseases. Previous studies have indicated that IR promotes the progression of CRLM (4), while the prognostic significance of IR in CRLM patients remains unclear. The gold standard for diagnosing IR is the “hyperinsulinemia-normal glucose clamp” method, but its application in clinical routine tests and large-scale cohort studies is greatly limited due to cumbersome operation, high cost, and strict technical requirements. In contrast, the triglyceride-glucose (TyG) index, a simple, feasible, and reliable alternative marker for IR (5), has offered greater clinical applicability. Despite its potential, research on the TyG index in oncology remains limited, and existing evidence is inconclusive (6–12). While most studies suggest that a high TyG index correlates with poor prognosis in various cancers, including CRC (6, 7), others report null or even opposite associations. For example, a large multi-center study reported no association between TyG and the risk of endometrial, ovarian, or postmenopausal breast cancer (13). Another study found that TyG was not related to prostate cancer mortality but was negatively correlated with its incidence (14). In lung cancer, the association between TyG and risk also remains controversial (15). Interestingly, the prognostic direction of TyG may vary by disease stage. Compared to early-stage disease, advanced pancreatic cancer shows decreased TyG levels (16). Furthermore, a retrospective study suggests low TyG level is associated with an increased risk of liver metastasis in pancreatic cancer, initially speculating that cachexia and the hepatic microenvironment influence TyG levels (17). Collectively, these findings underscore the need for further investigation, particularly regarding the prognostic role of the TyG index in CRLM, which remains largely unexplored.

Recently, machine learning (ML) has emerged as a powerful statistical tool capable of handling complex data characteristics, such as collinearity and non-linear relationships, which are often challenging for conventional methods. Its application in selecting variable, building clinical prediction models and assessing prognosis has therefore attracted growing interest (18).

As the liver is a core metabolic organ, metastatic infiltration may disrupt glucose and lipid homeostasis, potentially leading to distinctive TyG index levels in CRLM patients. Thus, we hypothesized a “paradoxical,” inverse association between the TyG index and overall survival in CRLM: lower TyG predicts poorer survival, contrary to the pattern typically seen in other cancer populations. Leveraging a large single-center retrospective cohort and combining traditional survival analysis with advanced ML algorithms, this study aimed to: (1). investigate the independent association between the TyG index and overall survival (OS) in CRLM patients; (2) identify key clinicopathological variables influencing CRLM prognosis; (3) develop and validate a nomogram incorporating the TyG index; and (4) compare the predictive performance of this model with the established Clinical Risk Score (CRS) system (19) to evaluate its potential clinical utility.

## 2 Materials and methods

### 2.1 Study population and data collection

This single-center, retrospective, observational cohort study enrolled patients initially diagnosed with synchronous colorectal cancer liver metastases (liver metastases detected at the time of initial diagnosis of colorectal cancer) between 2013 and 2023 at the Sixth Affiliated Hospital of Sun Yat-sen University. All included patients received curative-intent treatment for both the primary tumor and liver metastases. The inclusion criteria were: (1) histopathologically confirmed colorectal cancer with radiologically verified synchronous liver metastases; (2) underwent curative resection of the primary tumor, and achieved “no evidence of disease” status for liver metastases through surgical resection and/or local ablation. Exclusion criteria comprised: (1) history of other concurrent malignancies; (2) death from non-oncological causes; (3) missing essential clinical information or follow-up data.

Data were collected from the hospital’s electronic medical records and picture archiving and communication systems, including: (1) Baseline demographics: sex, age, body mass index (BMI); (2) Primary tumor characteristics: location, T stage, N stage, lymphovascular invasion, perineural invasion, differentiation grade, Ki67 proliferation index, Her2 status, KRAS mutation status; (3) Pre-treatment baseline of serological biomarkers: CEA and CA19-9 level, triglycerides (TG, mg/dL), fasting blood glucose (FBG, mg/dL), platelets (PLT), absolute neutrophil count (Neu), absolute monocyte count (Mono), and absolute lymphocyte count (Lym); (4) Liver metastases features: number of metastases, maximum diameter, bilobar distribution; (5) Treatment and follow-up data: receipt of chemotherapy, overall survival (OS), recurrence-free survival (RFS). TyG index, Systemic Immune-inflammation Index (SII) and SIRI (Systemic Inflammation Response Index) were calculated according to the following formulas.


•TyG⁢ln⁢[TG*FBG/2]

•SII⁢Neu*Plt/Lym

•SIRI⁢Neu*Mono/Lym

### 2.2 Statistical analysis

#### 2.2.1 Baseline characteristics and data processing

Continuous variables with skewed distributions were presented as median with interquartile range (IQR) and compared using the Mann-Whitney U test. Categorical variables were expressed as frequencies (percentages) and analyzed using the chi-square test or Fisher’s exact test, as appropriate. Optimal cutoff values for continuous variables were determined using the “survminer” R package based on survival outcomes, yielding the following thresholds for dichotomization: TyG index (8.311), age (57 years), BMI (22.76 kg/m^2^), SII (917.539), SIRI (0.521), CEA (14.79 ng/mL), CA19-9 (56.99 U/mL), number of liver metastases (2), and maximum diameter of liver metastases (2.8 cm).

#### 2.2.2 Survival analysis and variable screening

Survival curves were generated using the Kaplan-Meier method and compared with the Log-rank test. Univariate Cox proportional hazards regression analyses were initially conducted. Variables achieving a significance level of *p* < 0.05 were subsequently incorporated into a multivariate Cox regression model. Results are presented as hazard ratios (HR) with 95% confidence intervals (CI).

#### 2.2.3 ML model construction and variable importance

To address multicollinearity and identify the most informative predictors, Least Absolute Shrinkage and Selection Operator (LASSO) regression was applied with 10-fold cross-validation, selecting the optimal penalty parameter (λ) based on the minimum partial likelihood deviance. Variables with non-zero coefficients at the optimal λ were retained for subsequent machine learning modeling.

The dataset was randomly split into a training set (70%) and an internal validation set (30%). Using the LASSO-selected variables, six machine learning algorithms–Extreme Gradient Boosting (XGBoost), Partial Least Squares Discriminant Analysis (PLS), Random Forest (RF), Support Vector Machine (SVM), Logistic Regression (LR), and Naïve Bayes (NB)–were trained on the training set. Hyperparameters for each model were optimized via 10-fold cross-validation on the training set to prevent overfitting. Model discriminative performance was evaluated on the validation set using the area under the receiver operating characteristic curve (AUC) with 95% CIs.

To enhance model interpretability, the SHapley Additive exPlanations (SHAP) framework was employed to quantify each variable’s contribution to the predictions, providing both feature importance ranking and the directionality of effects. Variables consistently ranked among the top 10 across all six ML models were defined as the core variable set for subsequent prognostic model development.

#### 2.2.4 Nomogram development and validation

For nomogram development, the dataset was independently randomly partitioned into a training set (70%) and an internal validation set (30%). Based on the core variable set identified through the ML approach, a nomogram was developed on the training cohort via Cox proportional hazards regression. The model’s performance was assessed in the validation cohort using the C-index and time-dependent AUC values. Calibration was visually inspected using calibration curves. The predictive performance of our nomogram was further compared to that of the traditional Fong Clinical Risk Score (CRS).

## 3 Results

### 3.1 Patients characteristics

A total of 644 patients with synchronous CRLM who received curative-intent treatment were included in this study. The median follow-up time was 2.81 years (IQR: 1.85–4.23 years), during which 245 patients (38.0%) died. As summarized in Table 1, compared to survivors, patients in the deceased group exhibited significantly higher rates of early recurrence (38.37% vs. 12.78%, *p* < 0.001), a greater proportion of high-risk CRS scores (26.12% vs. 9.52%, *p* < 0.001), more advanced pN stage (78.78% vs. 57.39%, *p* < 0.001), and a heavier liver metastatic burden (>2 metastases: 42.45% vs. 31.58%, *p* = 0.005).

**TABLE 1:** Baseline characteristics and comparative analysis.

Variables	Total (*n* = 644)	Survivor (*n* = 399)	Non-survivor (*n* = 245)	Statistic	*P*
Recurrence type, *n* (%)				χ^2^ = 70.44	<0.001
None	336 (52.17)	253 (63.41)	83 (33.88)	–	–
≤1 year	145 (22.52)	51 (12.78)	94 (38.37)	–	–
>1 year	163 (25.31)	95 (23.81)	68 (27.76)	–	–
Sex, *n* (%)				χ^2^ = 0.09	0.761
Male	420 (65.22)	262 (65.66)	158 (64.49)	–	–
Female	224 (34.78)	137 (34.34)	87 (35.51)	–	–
Age, *n* (%)				χ^2^ = 6.54	0.011
≤57	354 (54.97)	235 (58.90)	119 (48.57)	–	–
>57	290 (45.03)	164 (41.10)	126 (51.43)	–	–
BMI, *n* (%)				χ^2^ = 0.08	0.777
≤22.76	438 (68.01)	273 (68.42)	165 (67.35)	–	–
>22.76	206 (31.99)	126 (31.58)	80 (32.65)	–	–
CEA, *n* (%)				χ^2^ = 4.90	0.027
≤14.79	288 (44.72)	192 (48.12)	96 (39.18)	–	–
>14.79	356 (55.28)	207 (51.88)	149 (60.82)	–	–
CA199, *n* (%)				χ^2^ = 15.51	<0.001
≤56.99	455 (70.65)	304 (76.19)	151 (61.63)	–	–
>56.99	189 (29.35)	95 (23.81)	94 (38.37)	–	–
TyG, *n* (%)				χ^2^ = 3.21	0.073
Low	169 (26.24)	95 (23.81)	74 (30.20)	–	–
High	475 (73.76)	304 (76.19)	171 (69.80)	–	–
SII, *n* (%)				χ^2^ = 0.90	0.342
Low	522 (81.06)	328 (82.21)	194 (79.18)	–	–
High	122 (18.94)	71 (17.79)	51 (20.82)	–	–
SIRI, *n* (%)				χ^2^ = 3.31	0.069
Low	74 (11.49)	53 (13.28)	21 (8.57)	–	–
High	570 (88.51)	346 (86.72)	224 (91.43)	–	–
Chemotherapy, *n* (%)				χ^2^ = 11.90	<0.001
No	66 (10.25)	28 (7.02)	38 (15.51)	–	–
Yes	578 (89.75)	371 (92.98)	207 (84.49)	–	–
Location, *n* (%)				χ^2^ = 0.99	0.32
Left	510 (79.19)	311 (77.94)	199 (81.22)	–	–
Right	134 (20.81)	88 (22.06)	46 (18.78)	–	–
pT, *n* (%)				χ^2^ = 3.62	0.057
I and II	69 (10.71)	50 (12.53)	19 (7.76)	–	–
III and IV	575 (89.29)	349 (87.47)	226 (92.24)	–	–
pN, *n* (%)				χ^2^ = 30.72	<0.001
No	222 (34.47)	170 (42.61)	52 (21.22)	–	–
Yes	422 (65.53)	229 (57.39)	193 (78.78)	–	–
Differentiation, *n* (%)				χ^2^ = 4.19	0.041
Middle and high	530 (82.30)	338 (84.71)	192 (78.37)	–	–
Low	114 (17.70)	61 (15.29)	53 (21.63)	–	–
Cancer thrombus, *n* (%)				χ^2^ = 5.25	0.022
No	494 (76.71)	318 (79.70)	176 (71.84)	–	–
Yes	150 (23.29)	81 (20.30)	69 (28.16)	–	–
Neural infiltration, *n* (%)				χ^2^ = 0.98	0.322
No	433 (67.24)	274 (68.67)	159 (64.90)	–	–
Yes	211 (32.76)	125 (31.33)	86 (35.10)	–	–
Ki67 level, *n* (%)				χ^2^ = 14.57	<0.001
>0.5	283 (43.94)	152 (38.10)	131 (53.47)	–	–
≤0.5	361 (56.06)	247 (61.90)	114 (46.53)	–	–
Her2, *n* (%)				χ^2^ = 2.32	0.128
No	494 (76.71)	314 (78.70)	180 (73.47)	–	–
Yes	150 (23.29)	85 (21.30)	65 (26.53)	–	–
Kras mutation, *n* (%)				χ^2^ = 3.84	0.05
No	529 (82.14)	337 (84.46)	192 (78.37)	–	–
Yes	115 (17.86)	62 (15.54)	53 (21.63)	–	–
Number of live metastases, *n* (%)				χ^2^ = 7.81	0.005
≤2	414 (64.29)	273 (68.42)	141 (57.55)	–	–
>2	230 (35.71)	126 (31.58)	104 (42.45)	–	–
Site of liver metastases, *n* (%)				χ^2^ = 2.49	0.114
Single	380 (59.01)	245 (61.40)	135 (55.10)	–	–
Double	264 (40.99)	154 (38.60)	110 (44.90)	–	–
Maximum diameter of liver metastasis, *n* (%)				χ^2^ = 1.59	0.207
≤2.8	110 (17.08)	74 (18.55)	36 (14.69)	–	–
>2.8	534 (82.92)	325 (81.45)	209 (85.31)	–	–
CRS level, *n* (%)				χ^2^ = 31.37	<0.001
High	102 (15.84)	38 (9.52)	64 (26.12)	–	–
Low	542 (84.16)	361 (90.48)	181 (73.88)	–	–
Treatment of liver metastases, *n* (%)				χ^2^ = 2.97	0.227
Surgery	248 (38.51)	154 (38.60)	94 (38.37)	–	–
Ablation	327 (50.78)	196 (49.12)	131 (53.47)	–	–
Both	69 (10.71)	49 (12.28)	20 (8.16)	–	–

χ^2^, Chi-square test.

Notably, baseline TyG levels did not differ between groups. This discrepancy likely reflects confounding or suppression: the prognostic effect of low TyG may be masked by unfavorable factors (e.g., advanced pN stage, higher liver metastasis burden) in unadjusted analysis.

### 3.2 Association of TyG index with survival outcomes

**FIGURE 1:**
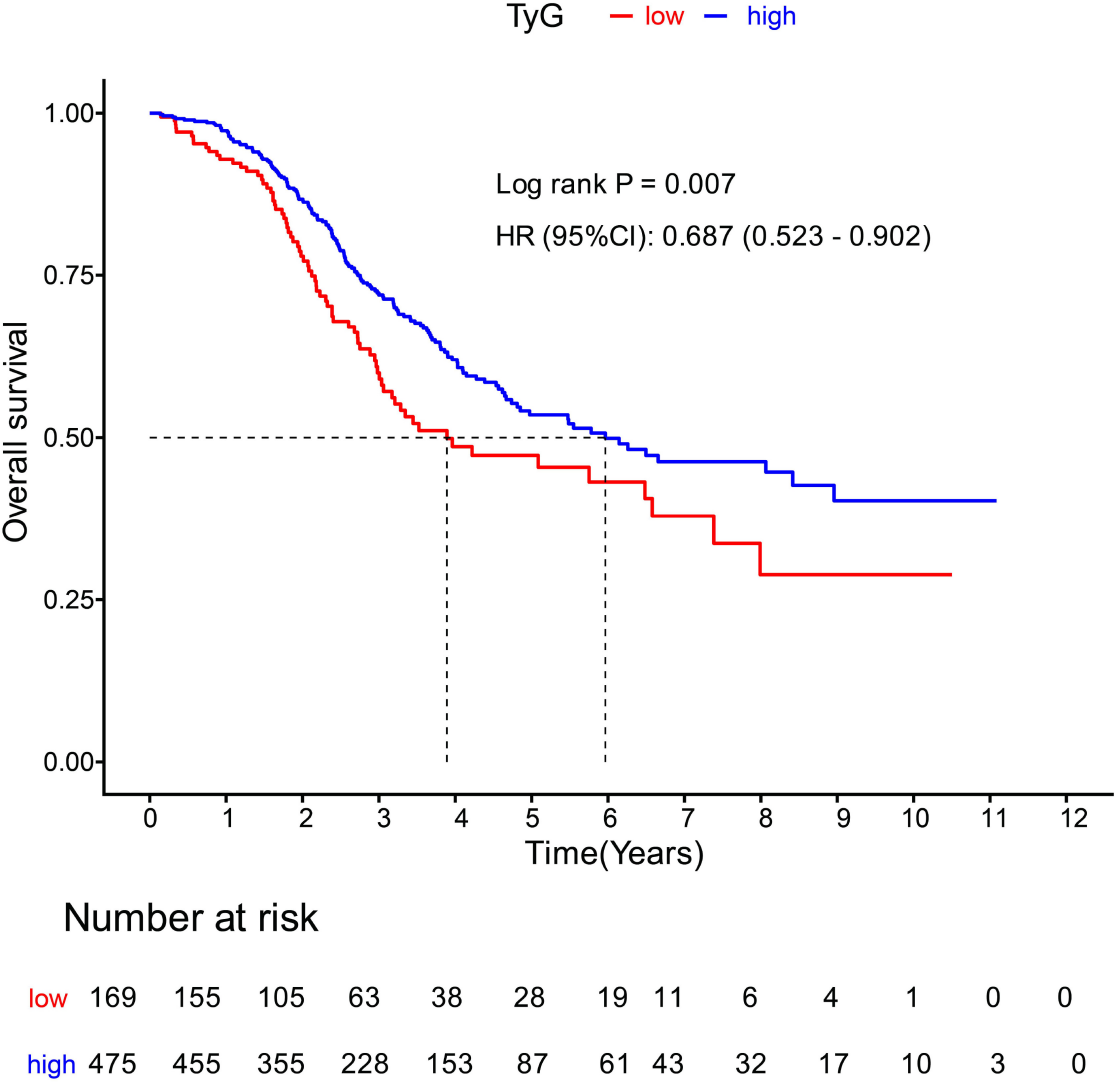
The Kaplan-Meier Curve for overall survival (OS) stratified by triglyceride-glucose (TyG) index. Patients with colorectal cancer liver metastases (CRLM) were stratified into low and high TyG index groups based on the optimal cutoff value (8.311). Survival curves were compared using the log-rank test. HR, hazard ratio; CI, confidence interval.

**FIGURE 2:**
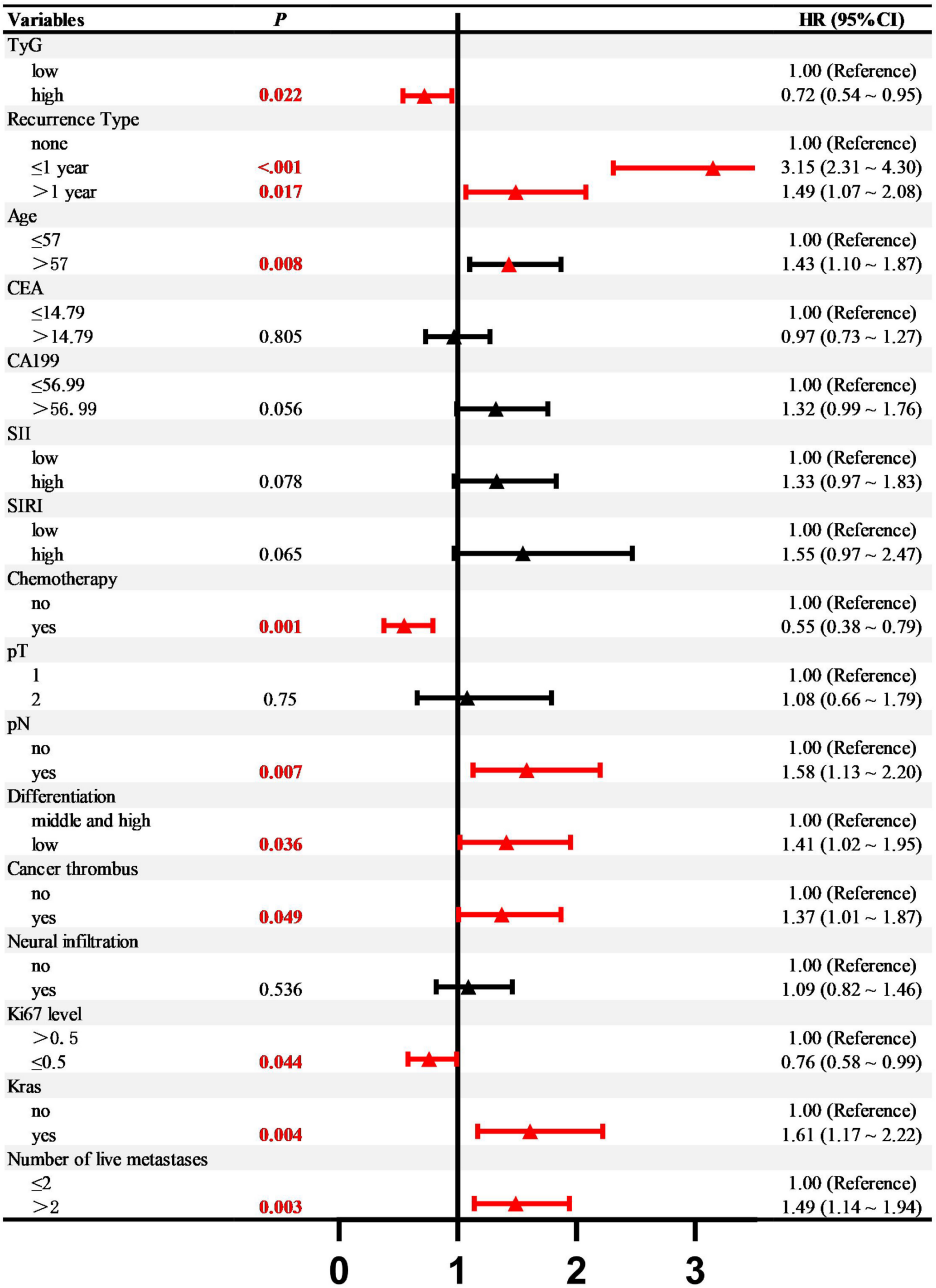
Multivariable cox regression analysis for overall survival (OS). Forest plot showing adjusted HR and 95% CI for variables included in the multivariable cox proportional hazards model. Variables with *p* < 0.05 were considered statistically significant. HR, hazard ratio; CI, confidence interval.

Kaplan-Meier survival analysis revealed that patients with a higher TyG index had significantly longer OS (HR = 0.687, 95% CI: 0.523–0.902; Log-rank *p* = 0.007) (Figure 1). This inverse association was corroborated by univariate Cox regression (HR = 0.69, 95% CI: 0.52–0.90, *p* = 0.007) (Supplementary Figure 1). After adjusting for potential confounders in the multivariate Cox analysis, a high TyG index remained independently associated with improved OS (HR = 0.72, 95% CI: 0.54–0.95, *p* = 0.022) (Figure 2). The robustness of this association was further supported through multi-model adjustment, where the significant relationship between low TyG index and mortality risk persisted after sequentially removing various covariate sets (Supplementary Figure 2).

### 3.3 Feature selection and ML model performance

To identify the most informative predictors, LASSO regression was initially performed, selecting 15 variables with non-zero coefficients from the initial pool: sex, age, CA19-9, SIRI, recurrence type, chemotherapy, pT stage, pN stage, tumor differentiation, cancer thrombus, KRAS mutation, HER2 status, Ki67 level, number of liver metastases, and maximum diameter of liver metastases (Supplementary Figures 3A, B).

**FIGURE 3:**
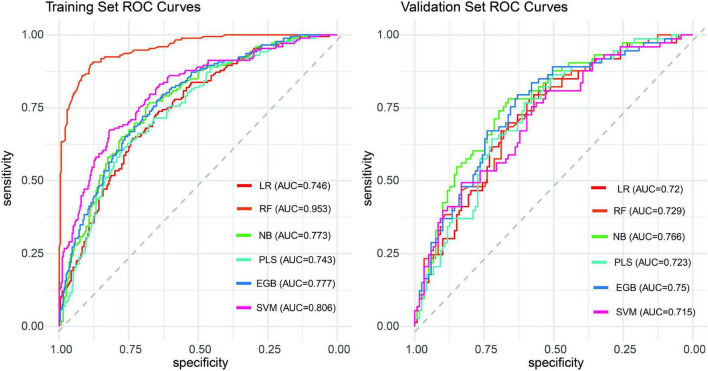
ROC curves comparing the discriminative performance of six machine learning models. LR, Logistic Regression. RF, Random Forest. NB, Naïve Bayes. PLS, Partial Least Squares Discriminant Analysis. EGB, Extreme Gradient Boosting. SVM, Support Vector Machine.

**FIGURE 4:**
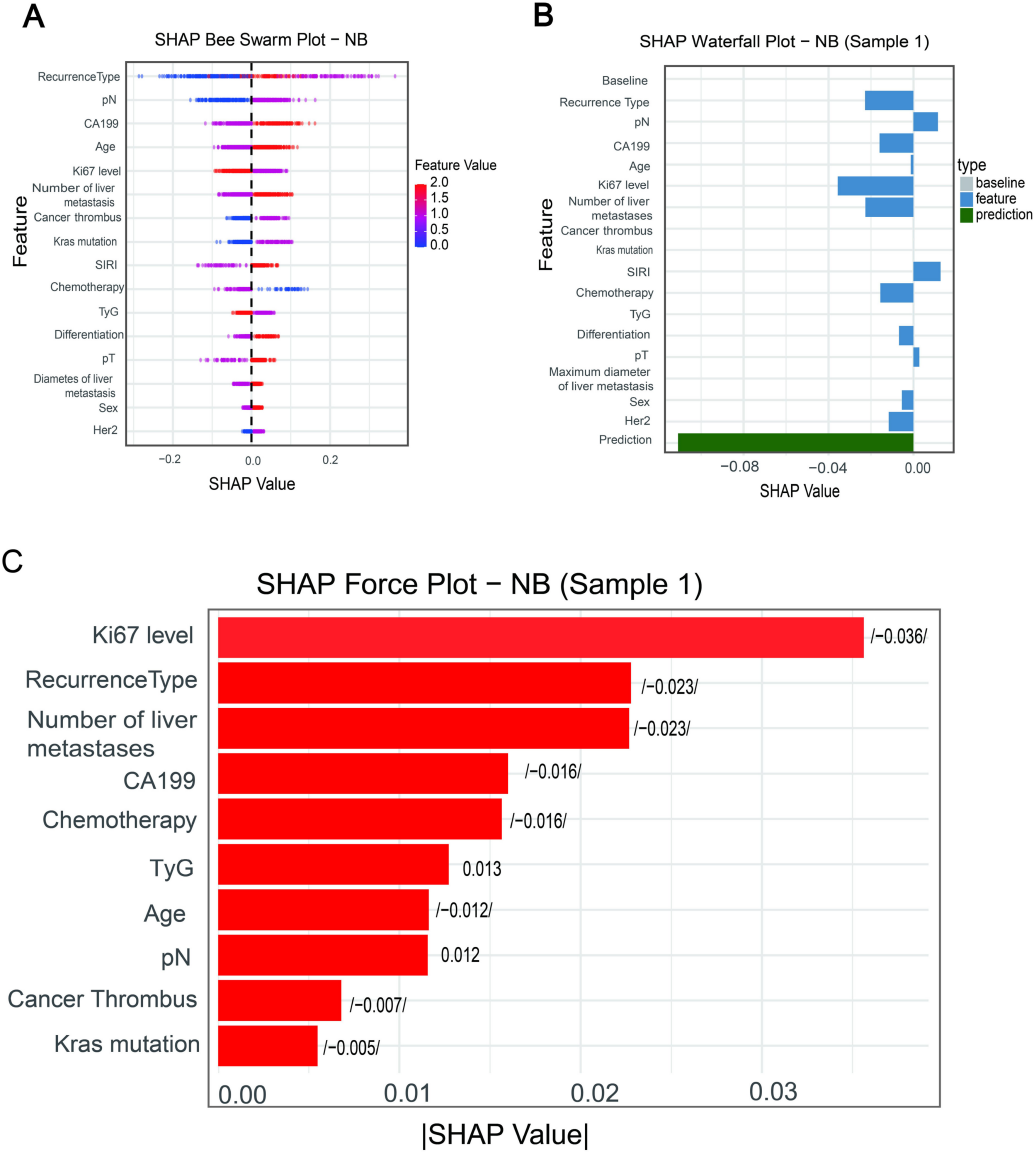
SHAP interpretation of the NB model. **(A)** Bee-swarm plot illustrating the distribution of SHAP values for each feature, with colors representing feature values (red, high; blue, low). Features are ordered by descending importance. **(B)** Waterfall plot showing the contribution of each feature to the model output for an individual patient. **(C)** Force plot visualizing the cumulative effect of features on the prediction for a representative case. SHAP, SHapley Additive exPlanations; NB, Naïve Bayes.

The cohort was randomly divided into a training set (*n* = 451) and an internal validation set (*n* = 193). Using the LASSO-selected variables along with TyG index, six machine learning models were constructed on the training set. When evaluated on the validation set, the Naïve Bayes (NB) model demonstrated the highest discriminative ability (AUC = 0.766, 95% CI: 0.698–0.835), outperforming the other five models (AUC range: 0.715–0.750) (Figure 3 and Supplementary Table 1). To enhance model interpretability, SHapley Additive exPlanations (SHAP) analysis was employed. For the top-performing NB model, the force plot revealed that Ki67 level, recurrence type, and number of liver metastases were the three most influential features driving predictions. Bee swam plot and waterfall plots provided consistent insights into variable importance and the direction of their effects (Figure 4). The force plot revealed that TyG index ranked in the top among all models, suggesting its predictive value for OS (Figure 4 and Supplementary Figure 4).

### 3.4 Development and validation of an integrated nomogram

By integrating feature importance rankings from all six ML models and identifying the intersection of consistently top-ranked variables, nine core predictors were selected for final model construction: TyG index, recurrence type, age, Ki67 level, CA19-9, number of liver metastases, pN stage, cancer thrombus, and KRAS mutation (Supplementary Figure 5).

**FIGURE 5:**
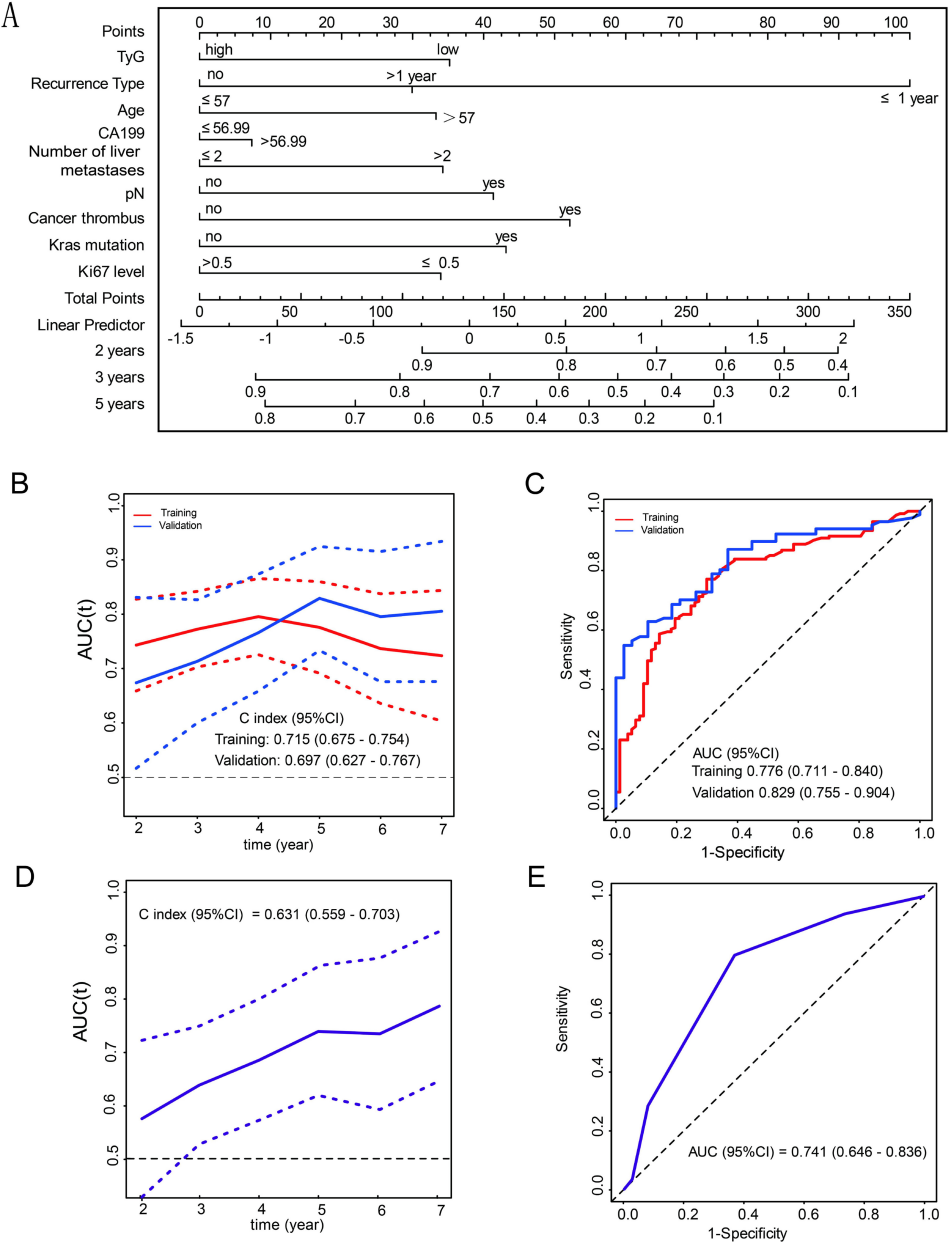
Development and validation of a prognostic nomogram for colorectal cancer liver metastases (CRLM). **(A)** Nomogram integrating nine core predictors for predicting 2-, 3-, and 5-year overall survival. **(B,D)** C-index curves comparing the predictive performance of the nomogram and the Fong Clinical Risk Score (CRS) over time. **(C,E)** ROC curves comparing the nomogram and CRS for 5-year overall survival prediction.

The original data were split into training and validation sets. A nomogram was subsequently constructed based on these nine variables using the training cohort (Figure 5A). For internal validation, the nomogram achieved a C-index of 0.697 (95% CI: 0.627–0.767) for predicting OS. Time-dependent ROC analysis demonstrated excellent predictive accuracy for 5-year survival, with an AUC of 0.829 (95% CI: 0.755–0.904) (Figures 5B, C). The risk score grouping derived from the nomogram could effectively distinguish the prognostic differences, and the risk score indicated predictive performance with AUC of 0.761, suggesting the robustness of this model (Supplementary Figure 6).

To assess clinical utility, the predictive performance of our nomogram was directly compared with the established Fong CRS model. Our model demonstrated better discrimination, with a higher C-index (0.697 vs. 0.631) and better AUC (5-year AUC: 0.829 vs. 0.741) (Figures 5B–E and Supplementary Figure 7).

The calibration curves demonstrated good agreement between predicted and observed survival probabilities. The DCA curve indicated the clinical net benefit (Supplementary Figure 7).

## 4 Discussion

By integrating conventional survival analysis with diverse ML approaches in a large CRLM cohort, our study yielded three principal findings. First, a lower TyG index—contrary to the commonly observed pattern—emerged as an independent risk factor for shortened OS in CRLM patients. Second, we successfully distilled a concise set of nine potent prognostic variables. Finally, the nomogram model constructed from these variables surpassed the traditional CRS in predicting OS.

### 4.1 “Paradoxical” role of TyG index in advanced CRLM

Our findings provide a novel perspective on the role of IR in cancer prognosis. Research on the TyG index in oncology remains limited, and existing evidence is inconclusive. Some prior research, spanning colorectal, breast, and other cancers, generally posits that an elevated TyG index (reflecting IR) is associated with tumor progression and inferior outcomes (6–11, 13). The prevailing mechanistic understanding posits that hyperinsulinemia, a consequence of insulin resistance, activates pro-survival signaling pathways such as PI3K/AKT/mTOR and NF-κB, thereby promoting cancer cell proliferation and survival (6, 20). However, a minority of studies have reported null or even contrary associations (13–15), and our results align with a limited but growing body of evidence in advanced-stage cancers, particularly those involving liver metastases (16, 17). Collectively, these observations suggest a potential context-dependent inverse association between TyG and prognosis in advanced disease.

### 4.2 Metabolic exhaustion and changes in liver function as potential mechanisms

The mechanism underlying the relationship between the TyG index and CRLM remains unclear. The following are possible theoretical explanations. This seemingly “paradoxical” association may be explained by the distinctive systemic and hepatic metabolic milieu characteristic of CRLM. Rather than reflecting metabolic health, a low TyG index in this setting likely signals a state of tumor-driven systemic metabolic exhaustion —a concept that can be understood through several interconnected mechanisms.

First, the “Warburg effect” provides a foundational framework (21, 22). In advanced CRLM, the substantial collective tumor burden acts as a potent “metabolic sink” (23), characterized by rampant glucose consumption via aerobic glycolysis. Concurrently, increased lipid mobilization depletes host triglyceride reserves to meet the energetic and biosynthetic demands of proliferating tumor cells (24, 25). Thus, a low TyG index may reflect the co-option and depletion of host glucose and lipid pools by the tumor.

Second, this paradigm of nutrient diversion is consistent with cancer cachexia—a syndrome prevalent in advanced disease. Cachexia paradoxically accelerates gluconeogenesis and lipolysis while consuming host energy stores (26–30), thereby suppressing circulating triglyceride levels and, consequently, the TyG index. The metabolic reprogramming characteristic of cachexia thus provides a complementary explanation for our observations.

Third, the liver’s central role in systemic metabolic homeostasis is directly compromised by metastatic replacement. Hepatic infiltration disrupts normal glucose and lipid regulation (31–33), potentially further lowering the TyG index. Moreover, liver dysfunction can elevate pro-inflammatory cytokines such as IL-6 and TNF-α, which in turn suppress hepatic triglyceride synthesis and secretion (34), creating a self-reinforcing cycle of metabolic dysregulation.

### 4.3 Methodological strengths and clinical implications

A key methodological strength of this study is the innovative integration of variable selection techniques. Moving beyond reliance on traditional Cox regression alone, we incorporated LASSO regression for initial feature refinement and then employed six distinct ML algorithms, using cross-validation and SHAP analysis for robust variable selection and interpretation. This approach enhanced model robustness and interpretability, clarifying the contribution and directionality of key predictors while keeping the final nomogram parsimonious.

From a clinical translation standpoint, our nomogram demonstrated favorable predictive performance. Its C-index and 5-year AUC exceeded those of the widely applied CRS. This suggests that a model integrating the TyG index alongside critical tumor biological features provides clinicians with a more precise tool for identifying high-risk patients who, despite undergoing curative-intent therapy, remain at elevated mortality risk, thereby potentially guiding intensified surveillance or adjuvant treatment strategies.

### 4.4 Limitations and future directions

Several limitations of this study should be acknowledged. First, the single-center retrospective design inherently carries risks of selection bias, which may limit external validity, and the findings may not be generalizable to unresectable or advanced CRLM populations. Second, the TyG index was measured only at baseline, ignoring assessment of the prognostic impact of dynamic metabolic changes during treatment or disease progression. Third, we were unable to account for several factors that may influence TyG index levels, including dietary status, lipid-lowering medications, and comorbidities such as diabetes mellitus. Fourth, while our ML approach enhanced predictive performance, the underlying biological mechanisms inferred from these associations remain speculative and warrant direct investigation. Additionally, data processing may introduce overfitting, reduce external reproducibility and increase the risk of model optimism due to multiple variable selection and repeated testing steps.

Future research should prioritize multi-center prospective validation to establish the generalizability of our model. Additionally, integrating multi-omics data—including metabolomics, radiomics, and inflammatory markers—could yield deeper biological insights and further refine predictive accuracy. Serial monitoring of metabolic indices such as the TyG index throughout the treatment course may reveal dynamic changes with prognostic significance and could inform adaptive treatment strategies. Finally, mechanistic studies exploring the interplay between tumor metabolism, host systemic responses, and liver function in CRLM patients could elucidate the biological basis for the paradoxical TyG-prognosis association observed here.

## 5 Conclusion

In conclusion, this study reports an association between a lower TyG index and poor overall survival in CRLM. The ML-based nomogram performed better than CRS in internal validation, but these findings are hypothesis-generating and need external validation and mechanistic investigation.

## Data Availability

The original contributions presented in this study are included in the article/Supplementary material, further inquiries can be directed to the corresponding authors.
