# Supplementary material for: “Paradoxical” prognostic role of the TyG index and a novel machine learning-derived nomogram for colorectal cancer liver metastases
Source: Front Nutr. 2026 Jul 13;13:1842975. doi: 10.3389/fnut.2026.1842975 (PMC13402184; doi:10.3389/fnut.2026.1842975)
Supplement: Supplementary file 1 [file Image_1.pdf]

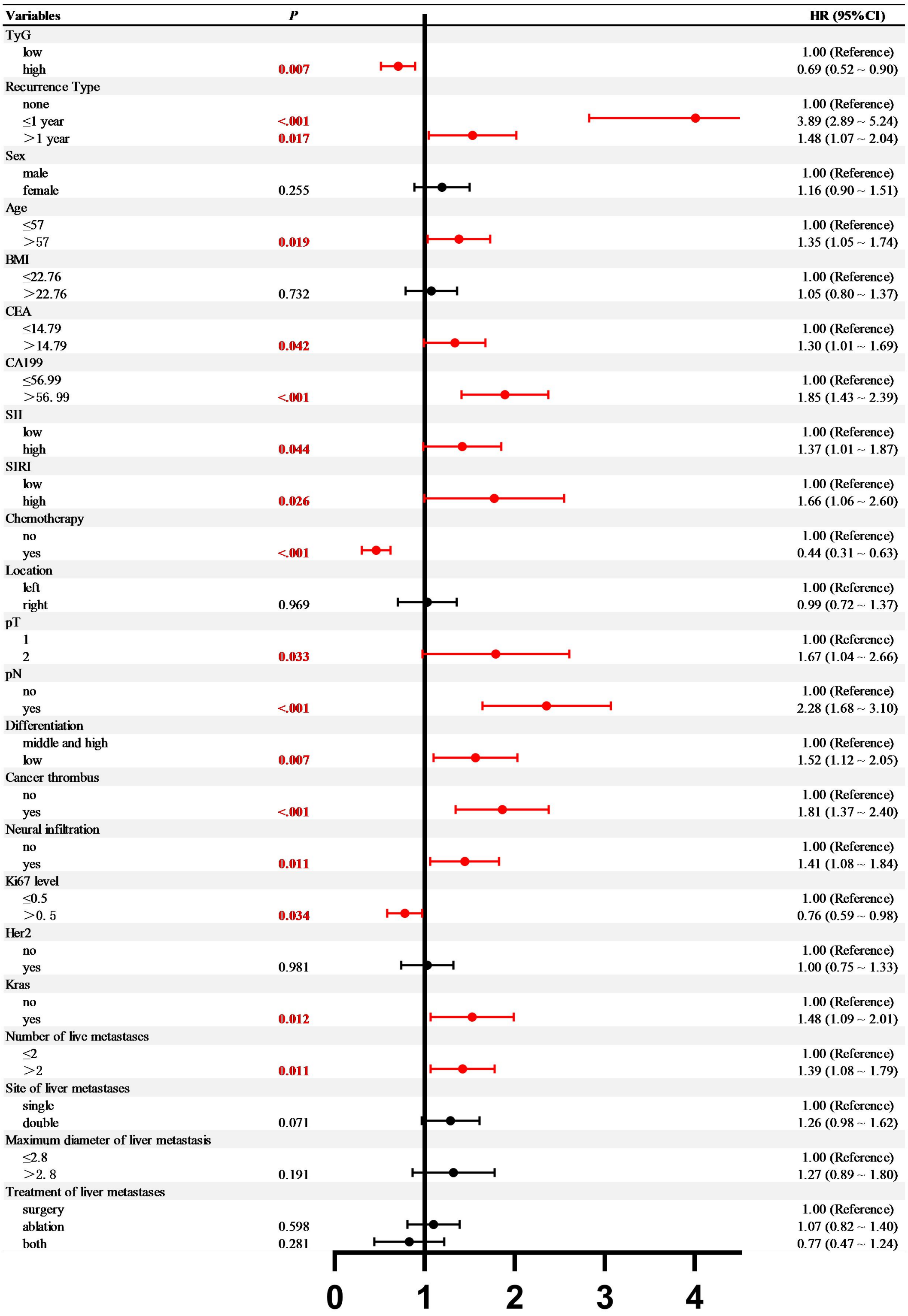

Fig.S 1: Univariate Cox Regression Analysis for OS. Forest plot displaying HR and 95% CI for all candidate variables in univariate Cox proportional hazards models. OS: Overall Survival. HR: hazard ratios. CI: confidence intervals.

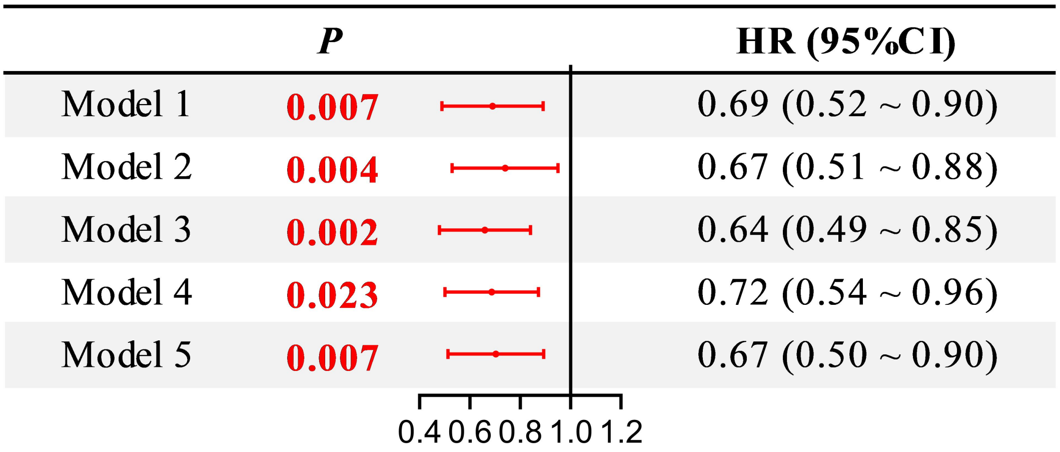

Model1: Crude

Model2: Adjust: Sex, Age, BMI

Model3: Adjust: Recurrence Type, Sex, Age, BMI, SII, SIRI

Model4: Adjust: Recurrence Type, Sex, Age, BMI, CEA, CA199, SII, SIRI, Chemotherapy, Location, pT, pN, Differentiation, Cancer thrombus, Neural infiltration, Ki67 level, Her2, Kras mutation

Model5: Adjust: Recurrence Type, Sex, Age, BMI, CEA, CA199, SII, SIRI, Chemotherapy, Location, pT, pN, Differentiation, Cancer thrombus, Neural infiltration, Ki67 level, Her2, Kras mutation, Number of live metastases, Site of liver metastases, Maximum diameter of liver metastasis, Treatment of liver metastases

Fig.S 2. Sensitivity Analysis of the Association Between TyG Index and OS. Forest plot demonstrating the stability of the TyG index–survival association after sequential adjustment for different covariate sets. The persistent protective effect of a higher TyG index supports the robustness of the findings. HR, hazard ratio; CI, confidence interval.

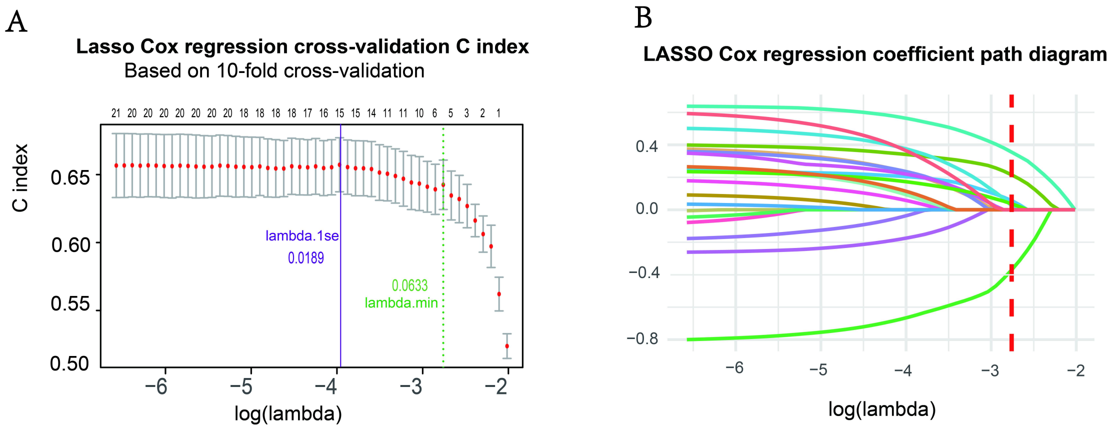

Fig.S 3: Feature Selection Using LASSO Regression. (A) Cross-validation plot for tuning parameter (λ) selection in LASSO regression. The optimal λ (lambda.1se = 0.0189) was chosen based on 10-fold cross-validation, resulting in the selection of 15 non-zero coefficient variables. (B) LASSO coefficient path plot showing the coefficient profiles of candidate variables as λ varies. LASSO, Least Absolute Shrinkage and Selection Operator.

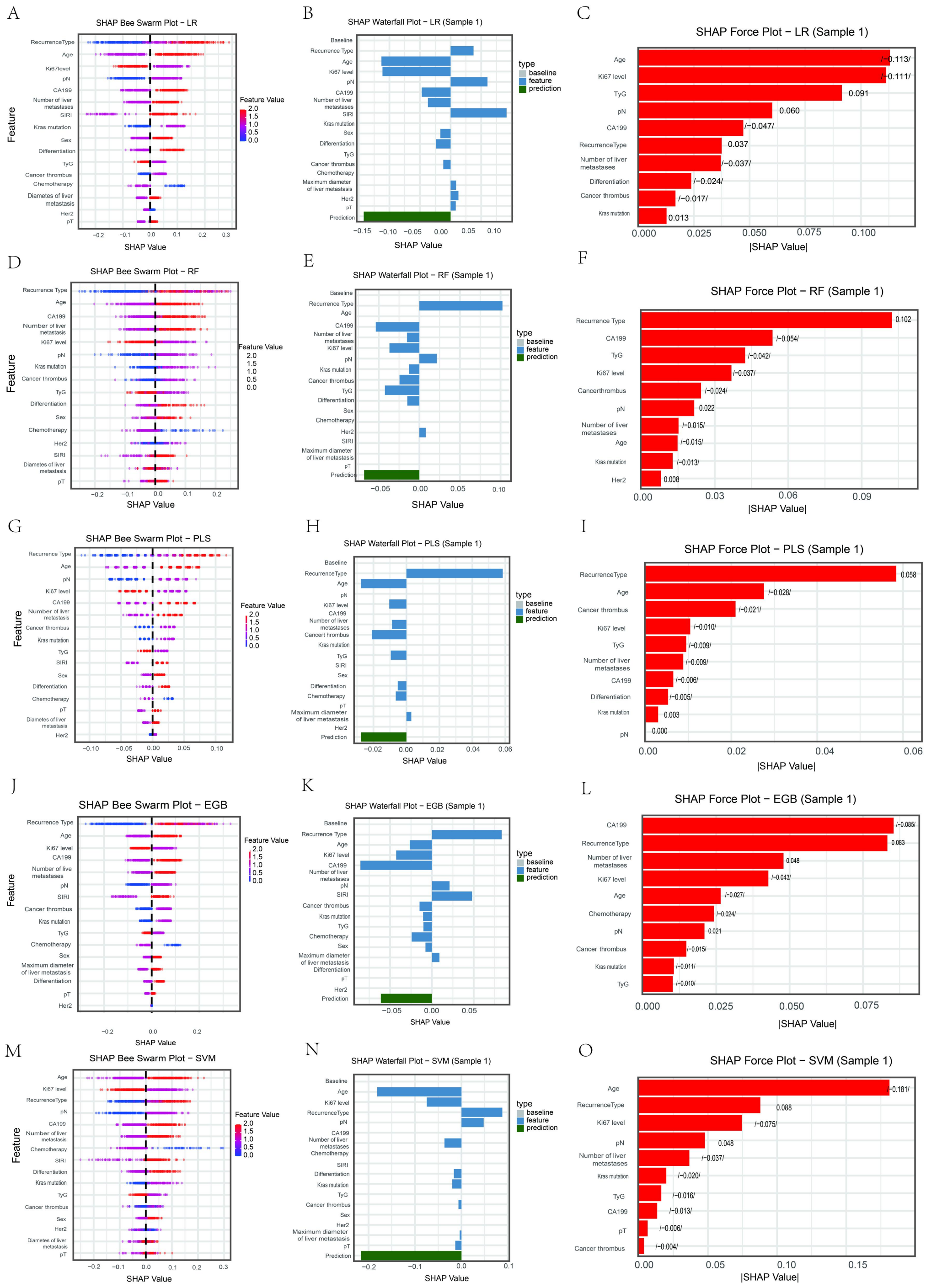

Fig.S 4: SHAP Interpretation for Five Machine Learning Models. SHAP bee-swarm plots, waterfall plots, and force plots for Logistic Regression (A–C), Random Forest (D–F), Partial Least Squares (G–I), Extreme Gradient Boosting (J–L), and Support Vector Machine (M–O). SHAP, SHapley Additive exPlanations; LR, Logistic Regression; RF, Random Forest; PLS, Partial Least Squares; XGBoost, Extreme Gradient Boosting; SVM, Support Vector Machine.

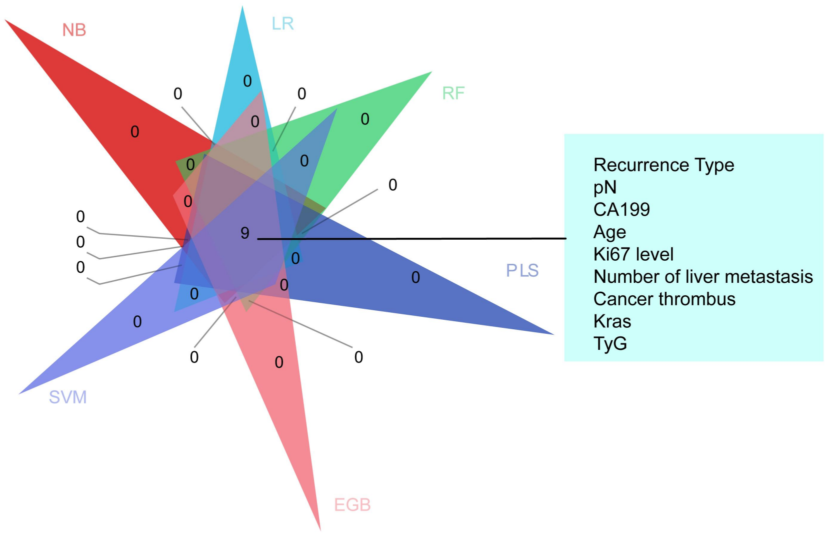

Fig.S 5: Identification of Core Predictor Set. Venn diagram illustrating the intersection of variables consistently ranked among the top 10 in feature importance across all six machine learning models. Nine core predictors were identified for final model construction.

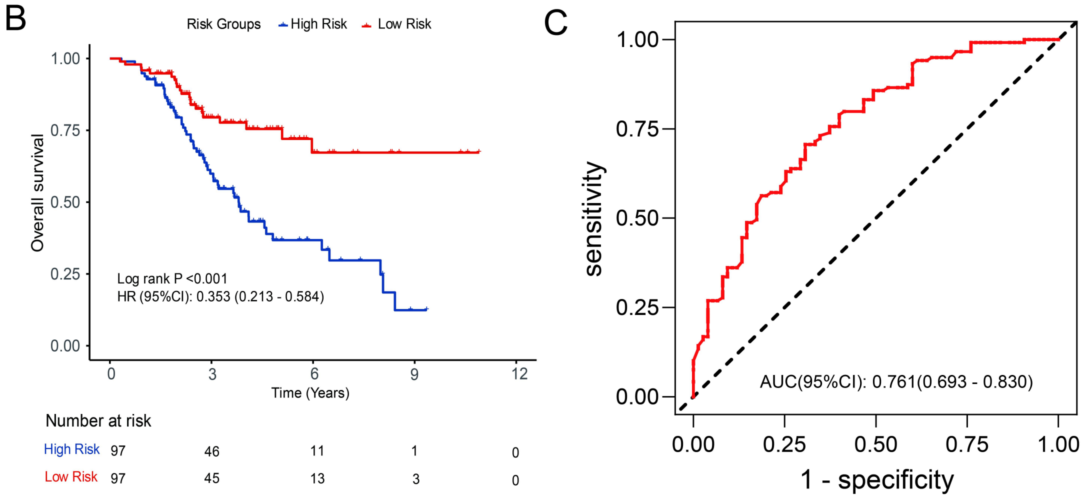

Fig.S 6: Kaplan-Meier Curve and ROC curve for Overall Survival Stratified by Risk Groups.

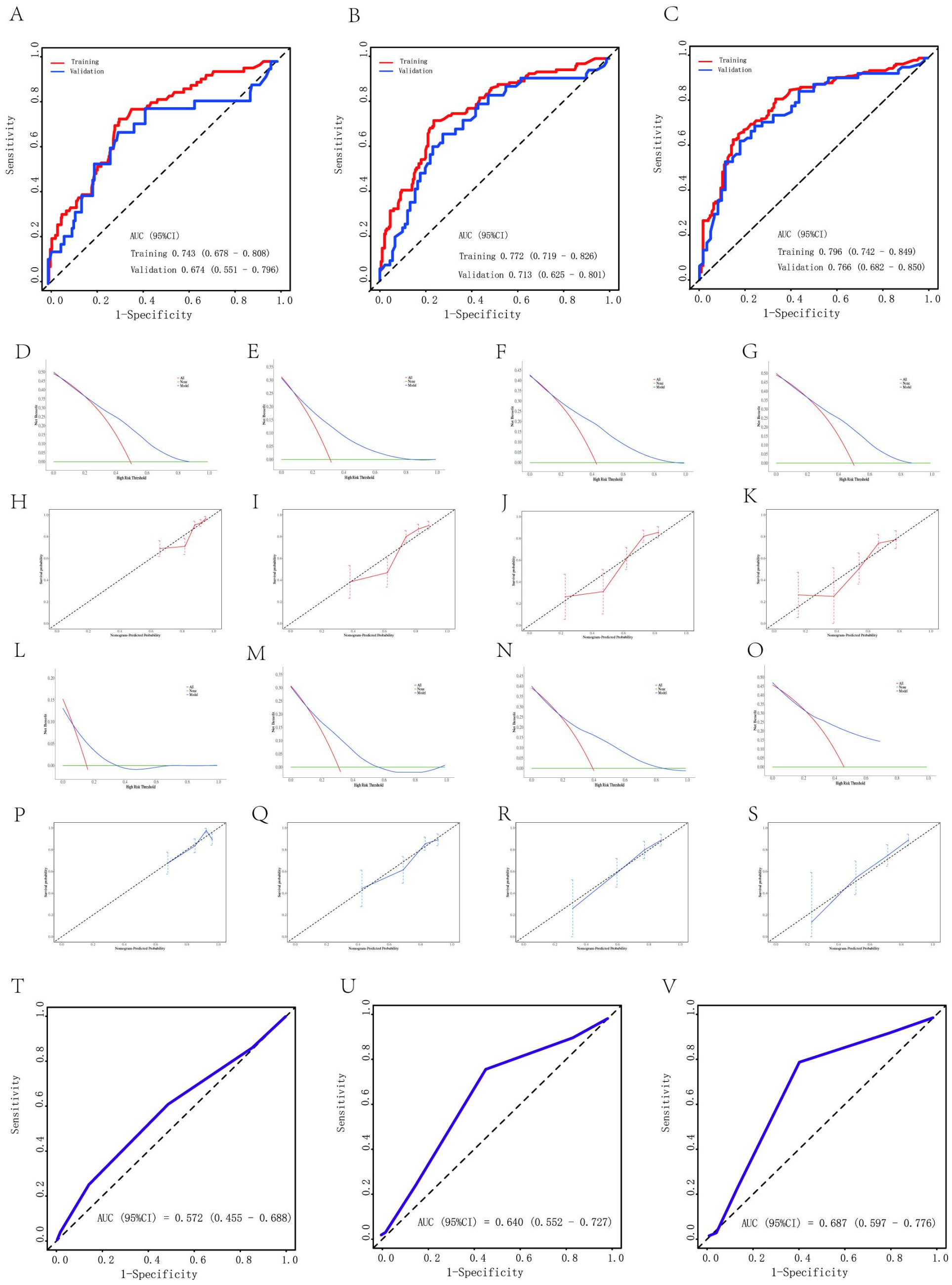

Fig.S 7: Performance Evaluation of the Nomogram and CRS. (A–C) Time-dependent ROC curves for the nomogram at 2, 3, and 4 years. (D–G) Decision curve analysis (DCA) for the nomogram at 2, 3, 4, and 5 years in the training cohort. (H–K) Calibration curves for the nomogram at 2, 3, 4, and 5 years in the training cohort. (L–O) DCA curves for the nomogram at 2, 3, 4, and 5 years in the validation cohort. (P–S) Calibration curves for the nomogram at 2, 3, 4, and 5 years in the validation cohort. (T–V) Time-dependent ROC curves for the Fong Clinical Risk Score (CRS) at 2, 3, and 4 years. ROC, receiver operating characteristic; DCA, decision curve analysis; CRS, Clinical Risk Score.
