# Supplementary material for: “Paradoxical” prognostic role of the TyG index and a novel machine learning-derived nomogram for colorectal cancer liver metastases
Source: Front Nutr. 2026 Jul 13;13:1842975. doi: 10.3389/fnut.2026.1842975 (PMC13402184; doi:10.3389/fnut.2026.1842975)
Supplement: Supplementary file 2 [file Table_1.pdf]

Supplementary Table 1: Model performance metrics

Models	Accuracy	Prevalence	Sensitivity	Specificity	Precision	F1_Score	MCC	AUC	AUC_Lower_CI	AUC_Upper_CI	Miss_Rate
LR	0.672	0.380	0.411	0.832	0.600	0.488	0.269	0.720	0.647	0.793	0.589
RF	0.661	0.380	0.247	0.916	0.643	0.356	0.224	0.729	0.658	0.801	0.753
NB	0.667	0.380	0.233	0.933	0.680	0.347	0.239	0.766	0.698	0.835	0.767
PLS	0.667	0.380	0.370	0.849	0.600	0.458	0.251	0.723	0.651	0.796	0.630
EGB	0.688	0.380	0.438	0.840	0.627	0.516	0.306	0.750	0.679	0.820	0.562
SVM	0.682	0.380	0.411	0.849	0.625	0.496	0.291	0.715	0.640	0.789	0.589
